# An investigation of dendritic delay in octopus cells of the mammalian cochlear nucleus

**DOI:** 10.3389/fncom.2012.00083

**Published:** 2012-10-22

**Authors:** Martin J. Spencer, David B. Grayden, Ian C. Bruce, Hamish Meffin, Anthony N. Burkitt

**Affiliations:** ^1^NeuroEngineering Laboratory, Department of Electrical and Electronic Engineering, University of MelbourneMelbourne, VIC, Australia; ^2^National ICT AustraliaMelbourne, VIC, Australia; ^3^Centre for Neural Engineering, University of MelbourneVIC, Australia; ^4^Bionics InstituteEast Melbourne, VIC, Australia; ^5^Department of Electrical and Computer Engineering, McMaster UniversityHamilton, ON, Canada

**Keywords:** auditory, cochlear nucleus, computational model, connectivity, octopus cells

## Abstract

Octopus cells, located in the mammalian auditory brainstem, receive their excitatory synaptic input exclusively from auditory nerve fibers (ANFs). They respond with accurately timed spikes but are broadly tuned for sound frequency. Since the representation of information in the auditory nerve is well understood, it is possible to pose a number of questions about the relationship between the intrinsic electrophysiology, dendritic morphology, synaptic connectivity, and the ultimate functional role of octopus cells in the brainstem. This study employed a multi-compartmental Hodgkin-Huxley model to determine whether dendritic delay in octopus cells improves synaptic input coincidence detection in octopus cells by compensating for the cochlear traveling wave delay. The propagation time of post-synaptic potentials from synapse to soma was investigated. We found that the total dendritic delay was approximately 0.275 ms. It was observed that low-threshold potassium channels in the dendrites reduce the amplitude dependence of the dendritic delay of post-synaptic potentials. As our hypothesis predicted, the model was most sensitive to acoustic onset events, such as the glottal pulses in speech when the synaptic inputs were arranged such that the model's dendritic delay compensated for the cochlear traveling wave delay across the ANFs. The range of sound frequency input from ANFs was also investigated. The results suggested that input to octopus cells is dominated by high frequency ANFs.

## Introduction

An important problem in neuroscience is to understand how neural function and processing are related to properties of the neuron, such as intrinsic electrophysiology, dendritic morphology, and patterns of synaptic innervation (Bock et al., 2011; Briggman et al., 2011; Seung, 2011). In our study, this relationship is investigated in octopus cells of the mammalian auditory brainstem using a computational modeling approach.

Octopus cells, located within the cochlear nucleus of mammals (Harrison and Irving, 1966; Osen, 1969), are known to respond with finely timed action potentials to acoustic onset events, such as the glottal pulses in speech (Godfrey et al., 1975; Rhode and Smith, 1986; Rhode, 1998). These sounds are characteristic of animal vocalizations, including speech and some environmental noise. Although these energy peaks are simultaneous across a broad range of frequencies, the transduction process in the cochlea introduces a differential delay. This is due to the “traveling wave delay” (Greenberg et al., 1997; Elberling et al., 2007; Ruggero and Temchin, 2007). The input to octopus cells from auditory nerve fibers (ANFs) tuned to low sound frequencies is delayed relative to the input from ANFs representing high sound frequencies. This raises the question of how octopus cells can respond with precise timing to broadband peaks in the temporal sound envelope, given that their input is temporally diffuse.

**Figure 1:**
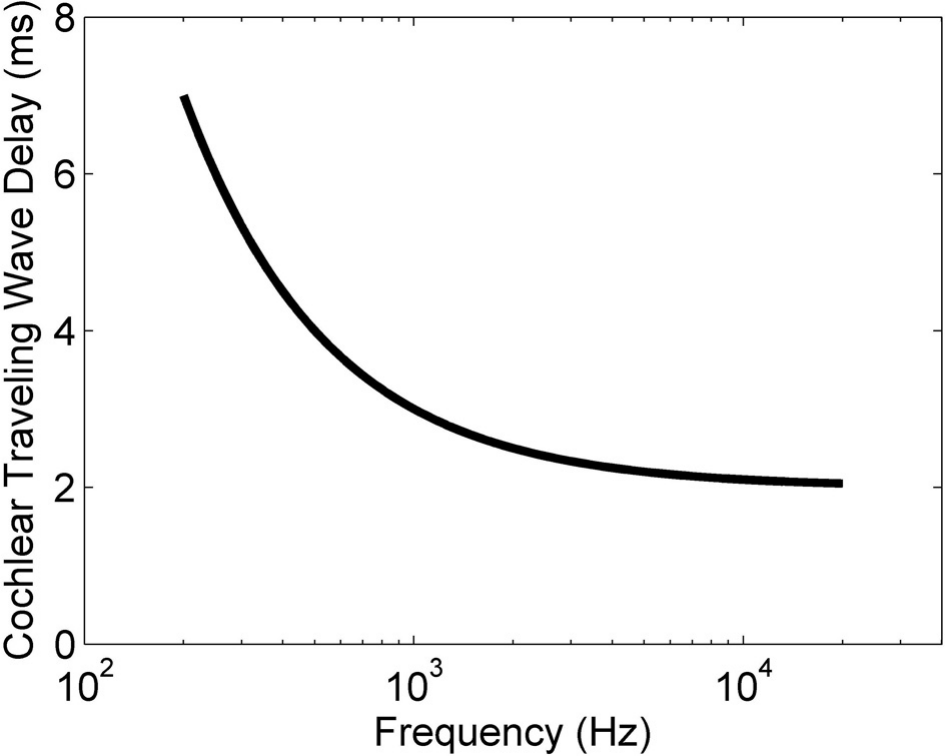
**The traveling wave delay in the basilar membrane of the cochlea of the cat as a function of ANF CF, as described by Equation (1).** In this case, *t*_offset_ = 2 ms.

The traveling wave delay is due to the mechanics of the transduction of sounds into neural signals by the cochlea. The cochlea receives input from the vibrations of the ossicles in the middle ear and provides output to the auditory nerve. Each ANF inherits a particular characteristic frequency (CF) by virtue of its location along the length of the Organ of Corti. The lowest CF ANFs respond with a latency of the order of many milliseconds longer than the highest CF ANFs. This tonotopic latency is inversely related to the ANF CF according to the approximate relationship (Greenberg et al., 1997)

(1) $$t_{\text{delay}}=(1000/f_{\text{CF}})+t_{\text{offset}},$$

where *t*_delay_ is the traveling wave delay measured in milliseconds, *f*_CF_ is the CF of a particular ANF measured in Hz, and *t*_offset_ is an offset time that depends on the particular experimental setup. This relationship was developed for the cat cochlea, but all mammals have delays of similar magnitudes (Ruggero and Temchin, 2007). Figure 1 shows the relationship between ANF CF and the traveling wave delay as described in Equation (1), using the value of *t*_offset_ from Greenberg et al. (1997) (2 ms). For the purposes of the present study we define the differential traveling wave delay (DTWD) as the difference in traveling wave delay between any two ANFs with different CFs. This quantity is more important than the absolute delay [described by Equation (1)] because the dendritic delay in octopus cells is hypothesized to compensate for the *difference* in the delays between synaptic inputs. For any two ANFs the DTWD can easily be calculated from Equation (1) by taking the difference between the two absolute traveling wave delays calculated from the two CFs.

Octopus cells are named after the monopolar morphology of their dendritic tree, consisting of approximately four or five wide-bore dendrites (see Table 1). This study examines the possibility that the dendrites of octopus cells provide a delay that compensates for the cross-frequency delay introduced by the traveling wave delay in the cochlea (Golding et al., 1999). Many experiments, though not all, have found that the dendrites of octopus cells course perpendicularly across the fibers of the auditory nerve (Willott and Bross, 1990; Oertel et al., 2000). In this orientation, in many mammalian species, the ANFs encoding high frequency sounds tend to synapse more distally than those representing lower frequency sounds. This anatomical organization is suggestive of the possibility that the dendritic delay in octopus cells provides compensation for the traveling wave delay. The possibility that dendritic delay might compensate for systematic asynchrony of synaptic input has been explored in the past [see, for instance, Agmon-Snir and Segev (1993) and Branco et al. (2010)]. These studies showed that dendritic delay is important in neurons that process more slowly varying synaptic input. However, the anatomical specializations of octopus cells, such as thick dendrites and a high density of voltage-gated channels, are predicted to significantly influence the time scale over which dendritic delays can influence neuronal computation.

**Table 1:** **Morphologies of octopus cells**.

**Species**	**Soma diameter (μm)**	**Axon diameter (μm)**	**Dendrite length (μm)**	**Dendrite width (μm)**	**Branches**	**Publications**
Cat	30	2	130	Irregular 1–7	2–3	Kane, (1973, Figure 6)
	30–40	3	300	Taper 3–8	6–7	Bal and Baydas, (2009, Figure 2)
	20–30	<5	350	Irregular 2–7	3–5	Smith et al., (2005, Figure 7)
	30	4	300	Irregular 3–8	3–5	Rhode et al., (1983, Figure 2)
Mouse	20–30	1.5	200	Irregular 0.5–4	4–6	Golding et al., (1995, Figure 3)
	15–35	1.5	100	Taper 0.5–3	5–6	Oertel et al., (1990, Figure 8)
Dog	35	3	250	Taper 2–8	3–4	Bal et al., (2009, Figure 2)

All values are approximate because they are taken from a direct examination of the published figures cited in the final column.

Octopus cells receive input directly from at least 60 ANFs, together representing a wide range of frequencies (Oertel et al., 2000). Their frequency tuning curves show two regions of greatest sensitivity: the first is situated at ~800 Hz, and the second between 2 and 20 kHz (Godfrey et al., 1975; Rhode and Smith, 1986). Furthermore, these two regions are associated with two different responses to tones. Below 800 Hz, octopus cells generally produce an action potential in response to every cycle of the tone, and above 2 kHz, octopus cells produce a single action potential at the onset of the tone, with no subsequent spikes (Godfrey et al., 1975; Rhode and Smith, 1986). Click-train sound stimuli (Oertel et al., 2000) and auditory nerve shock stimuli (Golding et al., 1995) produce similar bimodal responses. Octopus cells have also been observed to synchronize to the fundamental component of vocal sound stimuli (Rhode, 1998).

The behavior of octopus cells emerges as a result of the combination of their intrinsic electrophysiology, dendritic morphology and, importantly, their patterns of synaptic innervation. To investigate the interplay between these properties, we employed a computational model using published experimental results to constrain its parameters. Our results support the idea that the dendrites of octopus cells provide a time delay that compensates for the asynchronous arrival of auditory information across the tonotopy of ANFs. This compensation partly depends on the properties of low-threshold potassium channels located in the dendrites.

## Methods

The investigation proceeded in four phases. First, the model framework was established. Second, the model's parameters were constrained either directly, by using published experimental data, or indirectly, by modifying the model's parameters until its behavior matched that of published observations. Third, a number of hypotheses about the function of octopus cells and their component parts were investigated. Finally, the tolerance of the model's behavior to changes in its parameters was explored.

### Establishing the model framework

**Figure 2:**
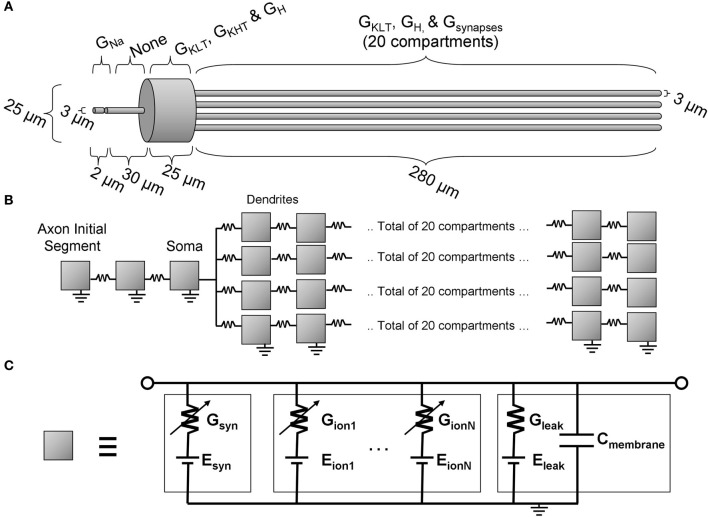
**The octopus cell model. (A)** The morphology with an indication of the locations of active ion channels. **(B)** The model topology showing the locations of each compartment (illustrated as a gray box). **(C)** The equivalent circuit representation of each compartment.

The multi-compartmental Hodgkin-Huxley model used in this study is schematically illustrated in Figure 2. The model is an extension of previous published models, principally Cai et al. (2000) and Rothman and Manis (2003b), although the present model is additionally equipped with an axon initial segment. The software package “NEURON” was used to implement and investigate the model (Carnevale and Hines, 2006). The time step of the model (*dt*) was set at 25 μs. The dendritic compartment resolution (*dx*) was set at 12.5 μm. These values were chosen such that the running time of the simulations was sufficiently short, but not so large that the conclusions of the study would be qualitatively inaccurate. A quantitative change was found with reduction of the value of *dt*, and this is described in the description of the results.

Morphological dimensions of the model were set at nominal values chosen to conform with the range of experimental results summarized in Table 1. The ANF type, number, CF and dendrite innervation mode used in previous modeling studies is summarised in Table 4. Four dendrites (250 μm long and 3 μm in width) were connected to the soma (Figure 2A). The soma (25 μm diameter) was connected to an axon (3 μm diameter) with an “initial segment.” The distance and length of the axon initial segment remains unconstrained by experiment, and so nominal values for these parameters were chosen (see Table 3). Sodium channels were placed in the axon initial segment (Kole et al., 2008; Clark et al., 2009; Fried et al., 2009; Kuba and Ohmori, 2009).

The model cell's membrane had a passive conductance and capacitance, ion channels were represented as a voltage-dependent conductance, and synapses were modeled as a conductance with a double-exponential temporal waveform. Coupled Hodgkin-Huxley equations governed the time-evolution of the membrane potentials in the compartments. Active ion channels were included to match the results of experimental studies. In addition to the standard sodium channels, the following ion channels were included in the model:

- Low-threshold potassium channels (514 ± 135 nS Bal and Oertel, 2001). Fluorescent markers have shown that these channels are present in octopus cells in the soma, dendrites, and also probably in the nodes of the axons (Rosenberger et al., 2003; Oertel et al., 2008).
- Hyperpolarization-activated mixed-cation channels (150 ± 30 nS) (Bal and Oertel, 2000). These channels have been found to be present in the soma and dendrites of octopus cells (Oertel et al., 2008).
- High-threshold potassium channels (116 ± 27 nS) (Bal and Oertel, 2001). These channels are likely to be present in the soma, since whole-cell patch clamp recordings detect their strong influence there. To date, fluorescent markers have not been used to study these channels' locations, although it has been shown that Kv3.1 are not in the soma (Perney and Kaczmarek, 1997). The channels are, therefore, likely to be type Kv3.3 (Li et al., 2001).

L-type calcium channels (Bal and Oertel, 2007) are most likely present in the dendrites and soma and, as they are calcium channels, may be important for synaptic plasticity. These channels were not included in the model as their long activation time constant and lack of voltage-dependent inactivation makes it unlikely that they are relevant for responses to input on the time scales investigated in this study.

Ion channels in the cell membrane of each compartment provide a voltage-dependent conductance. The membrane current for each compartment resulting from each channel-type is of the form

(2) $$I=G(a_{1}(t),a_{2}(t),\ldots )(V_{m}-E_{\text{reversal}}),$$

where *G* is the membrane conductance associated with the particular channel, and is a function of one or more gating variables *a*_1_, *a*_2_, etc., which are dependent on voltage and time, *V*_*m*_ is the membrane voltage, and *E*_reversal_ is the reversal potential associated with the particular channel. Leakage conductance in each compartment is passively dependent on voltage and can be described by considering *G* to be a constant.

The value of each gating variable is obtained through a numerical solution of the ODE

(3) $$\frac{da_{i}(t)}{dt}=\frac{Q}{a_{i\tau }(V_{m})}(a_{i\infty }(V_{m})-a_{i}(t)),$$

where *a*_*i*τ_(*V*_*m*_) and *a*_*i*∞_(*V*_*m*_) are channel-type specific functions of the membrane voltage. The variable *Q* is a temperature-dependent adjustment to the value of *a*_*i*τ_(*V*_*m*_) given by

(4) $$Q=Q_{10}^{(T-T_{0})/10},$$

where *T* is the temperature in degrees centigrade, *T*_0_ is the reference temperature (22°C), and *Q*_10_ = 3 (unless otherwise stated).

The sodium channel conductance was modeled using the implementation of Rothman et al. (1993). That model was, in turn, based on previous detailed experimental measurement (Frankenhaeuser and Huxley, 1964). This particular implementation of sodium channel dynamics has a rapid recovery from inactivation. The results of this study will depend, to some extent, on this particular model of sodium channel dynamics. The sodium conductance in each compartment evolves according to

(5) $$G_{Na}(m(t),h(t))=\bar{G_{Na}}m^{3}(t)h(t),$$

where $\bar{G_{Na}}$ is the maximum sodium conductance in that compartment, and *m* and *h* are the gating variables, where

(6) $$m_{\tau }(V_{m})\;=\frac{1}{-0.36(V_{m}+49){\left(e^{V_{m}+49/-3}-1\right)}^{-1}+\;0.4(V_{m}+58){\left(e^{V_{m}+58/20}-1\right)}^{-1}},$$

(7) $$m_{\infty }(V_{m})\;=\;\frac{1}{1+-1.11(V_{m}+58){\left(V_{m}+49\right)}^{-1}(e^{V_{m}+49/-3}-1){\left(e^{V_{m}+58/20}-1\right)}^{-1}}$$

(8) $$h_{\tau }(V_{m})\;=\frac{1}{2.4{\left(1+e^{(V_{m}+68)/3}\right)}^{-1}+0.8{\left(1+e^{V_{m}+61.3}\right)}^{-1}+3.6{\left(1+e^{-(V_{m}+21)/10}\right)}^{-1}},$$

(9) $$h_{\infty }(V_{m})=\frac{2.4{\left(1+e^{(v+68)/3}\right)}^{-1}+0.8{\left(1+e^{V_{m}+61.3}\right)}^{-1}}{2.4{\left(1+e^{(v+68)/3}\right)}^{-1}+0.8{\left(1+e^{V_{m}+61.3}\right)}^{-1}+3.6{\left(1+e^{-(V_{m}+21)/10}\right)}^{-1}}.$$

The low-threshold potassium channel model, available from the NEURON database (Carnevale and Hines, 2011), was derived by Rothman and Manis (2003a) from their experimental results:

(10) $$G_{K_{\text{LT}}}(w(t),z(t))=\bar{G_{K_{\text{LT}}}}w{\left(t\right)}^{4}z(t),$$

where

(11) $$w_{\infty }(V_{m})={\left(1+e^{-(V_{m}+48)/6}\right)}^{-0.25},$$

(12) $$w_{\tau }(V_{m})=100{\left(6e^{(V_{m}+60)/6}+16e^{-(V_{m}+60)/45}\right)}^{-1}+1.5,$$

(13) $$z_{\infty }(V_{m})=0.5+0.5{\left(1+e^{(V_{m}+71)/10}\right)}^{-1},$$

(14) $$z_{\tau }(V_{m})=1000{\left(e^{(V_{m}+60)/20}+e^{-(V_{m}+60)/8}\right)}^{-1}+50.$$

The high-threshold potassium channel model, available from the NEURON database (Carnevale and Hines, 2011), was also derived by Rothman and Manis (2003a) from their experimental results:

(15) $$G_{K_{\text{HT}}}(n(t),p(t))=\bar{G_{K_{\text{HT}}}}[0.85n^{2}(t)+0.15p(t)],$$

where

(16) $$n_{\infty }(V_{m})=\frac{1}{\sqrt{1+e^{-(V_{m}+15)/5}}},$$

(17) $$n_{\tau }(V_{m})=100{\left(11e^{(V_{m}+60)/24}+21e^{-(V_{m}+60)/23}\right)}^{-1}+0.7,$$

(18) $$p_{\infty }(V_{m})=\frac{1}{\left(1+e^{-(V_{m}+23)/6}\right)},$$

(19) $$p_{\tau }(V_{m})=\frac{100}{4e^{(V_{m}+60)/32}+5e^{-(V_{m}+60)/22}}+5.$$

The hyperpolarization-activated mixed-cation channel model, available from the NEURON database (Carnevale and Hines, 2011), was derived by Bal and Oertel (2000) from their experimental results:

(20) $$G_{I_{h}}(h(t))=\bar{G_{I_{h}}}h(t),$$

where

(21) $$h_{\tau }(V_{m})=\frac{125e^{10.44(V_{m}+50)/(273.16+T)}}{1+e^{34.81(V_{m}+50)/(273.16+T)}},$$

(22) $$h_{\infty }(V_{m})\;=\frac{1}{1+e^{(v+66)/7}}.$$

For the hyperpolarization-activated mixed-cation conductance, the values of the constants in Equation (4) were set to *T*_0_ = 33°C and *Q*_10_ = 4.5, as determined by Magee (1998).

The influence of each synapse was modeled as a conductance with a double exponential function,

(23) $$G_{\text{syn}}(t)=W*(e^{-t/\tau_{\text{decay}}}-e^{-t/\tau_{\text{rise}}}),$$

where τ_rise_ and τ_decay_ are the rise and decay time constants of the synaptic conductance, respectively, and *W* is an arbitrary weight that is used to adjust the magnitude of the contribution of a particular synapse. The ANF CFs were distributed logarithmically to match the cochlear frequency map. When connected to the model's dendrite, they were spaced linearly along its length. The strength of synapses along the dendrites were adjusted such that they had a uniform influence at the soma.

Synaptic input was provided using a simulation of the auditory periphery (Zilany and Bruce, 2006, 2007; Zilany et al., 2009) [the particular model used is that described in Zilany et al. (2009)]. This model has a middle-ear filter to give realistic responses to broadband acoustic signals, has realistic cochlear tuning properties (including time-varying and level-dependent changes in tuning), produces appropriate statistics of phase-locked spike times, and has physiologically-realistic group delay and phase characteristics as a function of cochlear position and acoustic signal level. It was validated using a range of sounds, including speech (Zilany et al., 2009). We showed that the periphery model accurately recreated the traveling wave delay by superimposing its output with the prediction of Equation (1).

### Parameter constraint and verification

The model parameters were selected to most accurately reflect the values expected in the cat. Each parameter of the model was determined in one of four ways:

1. By direct reference to the experimental evidence,
2. when measurement was not available, by adjustment to achieve experimentally realistic behavior of the model,
3. constrained by the experimental evidence but adjusted to investigate the effect of dendritic delay,
4. or left as a free parameter.

The third method was employed where experimental evidence was available but where dendritic delay may be dependent on variation in the parameter. The final method was used where there was no available direct or indirect experimental data. In Tables 2, 3, the parameters of the model are classified using these four categories.

**Table 2:** **Model parameter values that were set directly from experimental evidence presented in published papers**.

**Parameter**	**Value**	**References**
**DIRECTLY EXPERIMENTALLY CONSTRAINED**		
Model temperature	33°C/37°C	
Membrane capacitance	0.9 μF/cm^2^	
Passive membrane conductance	2 mS/cm^2^	Bal and Oertel, 2000; Bal and Baydas, 2009^a^
Passive membrane conductance reversal potential	−62 mV	Golding et al., 1999; Bal and Oertel, 2001
Axial resistivity	100 Ω.cm	
Synaptic reversal potential	0 mV	
PSP rise time constant	70 μ s	Gardner et al., 1999; Cao and Oertel, 2010^b^
PSP decay time constant	340 μ s	Gardner et al., 1999; Cao and Oertel, 2010^b^
Soma diameter	25 μm	See Table 1
Axon diameter	3 μm	See Table 1
Soma low-threshold potassium max conductance	40.7 mS/cm^2^	Bal and Oertel, 2001^c^
Soma high-threshold potassium max conductance	6.1 mS/cm^2^	Bal and Oertel, 2001^c^
Soma hyperpolarization-activated mixed-cation max conductance	7.6 mS/cm^2^	Bal and Oertel, 2000^c^
Low-threshold potassium conductance rev potential	−70 mV	Bal and Oertel, 2001; Rothman and Manis, 2003c
High-threshold potassium conductance rev potential	−70 mV	Bal and Oertel, 2001; Rothman and Manis, 2003c
Hyperpolarization-activated mixed-cation conductance rev potential	−38 mV	Bal and Oertel, 2000; Rothman and Manis, 2003c
Sodium conductance rev potential	55 mV	Frankenhaeuser and Huxley, 1964; Rothman et al., 1993

The values were selected to most accurately reflect the values expected in a cat. The model's temperature was set to 33°C for in vitro simulations and 37°C for in vivo simulations.

a The value ~2 mS/cm was calculated using the value of cell capacitance and a time constant of 0.5 ms. The references show an octopus membrane time constant of 0.2 and 1.3 ms, providing justification for the choice.

b These two publications provide different estimates of the synaptic time constants. The value listed in the table is an average of the two, after compensation is made for temperature.

c These studies quote a value for the total cell conductance. The value listed in the table, and the interpretation used for this study, is that this value of conductance is that of the soma only, and does not give an indication of dendritic conductance.

**Table 3:** **Model parameter values not directly and completely constrained by experimental evidence**.

**Parameter**	**Value**	**References**
**INDIRECTLY EXPERIMENTALLY CONSTRAINED**		
Axon initial segment sodium conductance	4244.1 mS/cm^2^	Ferragamo and Oertel, 2002; Bal and Baydas, 2009
		Frankenhaeuser and Huxley, 1964; Rothman et al., 1993^a^
Frequency span of synaptic innervation (Figure 13)	5.75–11 kHz	Golding et al., 1995; Godfrey et al., 1975
		Gardner et al., 1999; Cao and Oertel, 2010^a^
Frequency span of synaptic innervation (all others)	2.5–5 kHz	Golding et al., 1995; Godfrey et al., 1975
		Gardner et al., 1999; Cao and Oertel, 2010^a^
Number of synapses	300	Brown et al., 1990; Gardner et al., 1999
		Cao and Oertel, 2010^a^_,_^b^
**DIRECTLY EXPERIMENTALLY CONSTRAINED (LATER VARIED TO INVESTIGATE THEIR INFLUENCE ON DENDRITIC DELAY)**		
Synaptic conductance	2 nS	Gardner et al., 1999; Cao and Oertel, 2010^b^
Dendrite width	3 μm	See Table 1
Dendrite length	250 μm	See Table 1
**EXPERIMENTALLY UNCONSTRAINED (LATER VARIED TO INVESTIGATE THEIR INFLUENCE)**		
Passive axon segment length	10 μm
Axon initial segment length	20 μm
Dendrite low-threshold potassium conductance	2.7 mS/cm^2^
Dendrite hyperpolarization-activated conductance	0.6 mS/cm^2^

The titles indicate the methods that were used to select their value.

a The references include publications with both the experimental results and anatomical data that were used to constrain the parameter in the model.

b The value chosen is at the highest end of the experimental data.

**Table 4:** **ANF innervation in previously published Hodgkin-Huxley models of octopus cells**.

**Type of ANFs**	**Number of ANFs**	**CF range (kHz)**	**Mode**	**Publications**
High spontaneous rate	60	1.40–4.00	High to low CFs are proximal to distal	Levy and Kipke, 1997
High spontaneous rate	120	0.88–2.97	High to low CFs are distal to proximal	Cai et al., 2000
High spontaneous rate	60	1.40 only	Single compartment model only	Hemmert et al., 2005

The somatic concentrations of hyperpolarization-activated mixed-cation conductance and low-threshold potassium conductance in the model were fixed to match experimental results. In doing this, we do not wish to ignore the fact that, even in an individual animal, octopus cells will possess a spectrum of properties, with no two cells being the same (see “Discussion”). Variation of the order of 10% was explored to test the model's sensitivity, but this was not enough to account for the likely variation between cells. Since experiments have indicated the presence, but not the precise density of dendritic ion channels, the dendrite density was left as a free parameter in the model.

Unless stated otherwise, the synaptic weights, *W*, were manually adjusted to ensure that the influence of the synapse at the soma was independent of the synapse's position on the dendrite. This was done to ensure that it was the relative timing (rather than the relative size) of PSPs that would be the important factor determining the firing behavior of the model. The reversal potential, *E*_syn_, for the excitatory synaptic conductance, shown in Figure 2C, was set to 0 mV. Investigations of synaptic input in mice at 33°C (Gardner et al., 1999; Cao and Oertel, 2010) show a rise time of approximately 50–200 μs (Gardner et al., 1999; Cao and Oertel, 2010) and decay time of 350–800 μs (Gardner et al., 1999; Cao and Oertel, 2010). It was decided to take an average of 100 μs rise time and 500 μs decay time. These values were reduced by a factor of 1.5 to account for the temperature difference from 33°C to 37°C, giving a 70 μs rise time and 340 μs decay time. As suggested by experimental results (Liberman, 1993) and as in all previous computational investigations, only high spontaneous rate fibers were used as input. For the purposes of this model, it is taken that these are fibers that have a spike production rate of 50 Hz in the absence of auditory stimulus, and begin to increase their spike rate even at low sound intensity.

To determine the total synaptic drive to the model cell, we used experimental data relating to the number and strength of synaptic inputs, as well as observations of the sensitivity of the model cell. Using whole-cell patch-clamp techniques, Gardner et al. (1999) and Cao and Oertel (2010) computed an average peak synaptic conductance of 0.9 ± 0.45 nS and 1.5 nS, respectively. However, Cao and Oertel (2010) also observed PSPs that they calculated to be due to synapses of up to 2 nS. We considered this value to be the maximum limit, at the soma, for the value of this parameter of the model. Both Oertel et al. (2000) and Cao and Oertel (2010) suggest that the number of ANF inputs number more than 60, perhaps many times greater.

With model configuration (i) (shown in Figure 10), a parameter search for an appropriate synaptic drive was conducted. The appropriate synaptic drive was one that resulted in single action potential response to high frequency tones. It was found that 300 ANFs with 2 nS synapses provided a desirable, one action potential, response to 3 kHz tones of a variety of intensities. This frequency range of stimuli were chosen because they are commonly found in human speech.

Table 5 shows ANF innervation derived indirectly from an examination of the figures in published experimental studies. The published figures showed either the sound intensity threshold of octopus cells in response to tones of different frequencies or the spike rate in response to tones of differing intensities and frequencies. In each case, the octopus cell showed a double lobed, bimodal response curve. The bandwidth of ANF innervation was deduced from the width of the higher frequency lobe.

**Table 5:** **ANF innervation inferred from an examination of figures in previously published experimental investigations**.

**ANF CF range (kHz)**	**Publications**	**DTWD (ms)**
11–19	Rhode and Smith, (1986, Figure 3B)	0.04
1.5–3.0	Godfrey et al., (1975, Figure 8A)	0.33
5.0–14	Godfrey et al., (1975, Figure 8A)	0.13
20–40	Godfrey et al., (1975, Figure 8A)	0.03
4.5–11	Godfrey et al., (1975, Figure 15)	0.13

The inference requires the assumption that the high frequency lobe of octopus cells represents the frequency range of ANF input to those cells. Both papers investigated cats, and each row of the table deals with an individual octopus cell. The “ANF CF range” values in the first column of the table are used to calculate the DTWD values in the final column using Equation (1). The column marked DTWD, therefore, represents the dendritic delay required to compensate for the cochlear traveling wave delay present across the input ANFs.

The possibility that the dendritic delay compensates for DTWD requires the realistic periphery model, as well as a constraint of the frequency span of ANF innervation, since the DTWD is CF-dependent. To justify this constraint, the mean CF and frequency span of ANF innervation were explored to find a configuration that produced the most realistic response to sounds. Tones of different frequencies and intensities were used to characterize the model's response. This task was undertaken with different profiles of DTWD. Having obtained a clear candidate DTWD configuration, it was then this successful configuration that was used to test the model's response to speech, and to test the importance of dendritic delay.

### Tolerance of the model's behavior to parameter variation

The model's tolerance to parameter changes was investigated. A simple technique was used: parameters were individually varied by ±10% and the intrinsic excitability of the model was examined. Changes in behavior were classified as substantial if any of the following occurred:

1. There were missing or extra action potentials in response to a given step of current injection,
2. the model reverted to a conventional membrane voltage threshold response mode, rather than a membrane voltage rate-of-change threshold mode,
3. there was a change from a tonic (continuous) to phasic (onset) response (or vice-versa) for a given step of current injection, or a given tone stimulus,
4. or there was an observed change in some quantity by more than 10%, where this quantity might be the amplitude of the action potentials or the value of the rate-of-change of the membrane potential at which an action potential is produced, for example.

## Results

### Parameter constraint and verification

#### Model simulations indicate somatic voltage-clamp experiments underestimate the total conductance of low-threshold potassium channels

**Figure 3:**
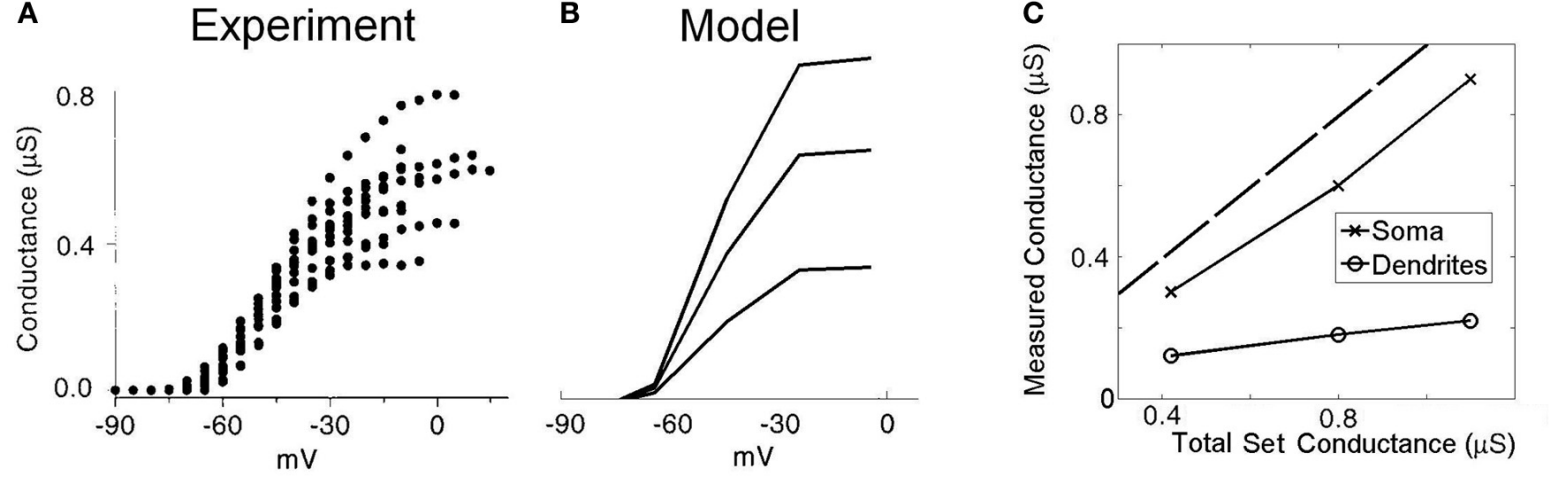
**Low-threshold potassium conductance as a function of membrane voltage. (A)** Experimental results showing voltage-clamp experiments for a number of real octopus cells. The conductance is calculated for a range of voltage-clamp levels. The maximum conductance is taken as the highest level of conductance (at the most depolarized voltages). **(B)** Model results with the low-threshold potassium channels located in both the dendrites and the soma as previously described. The three lines are associated with three different low-threshold potassium conductances in the soma, with maximum conductances 1.10, 0.80, and 0.42 μS. **(C)** Plot showing the measured conductance as a function of the total conductance of low-threshold potassium channels. The two curves show the result when the potassium channels are located entirely in either the soma or the dendrite. The broken line indicates the point of parity. The results in **(A)** are reproduced with permission from Bal and Oertel, (2001, Figure 5), Journal of Neurophysiology, Am Physiol Soc, used with permission.

Previous experimental investigations have used whole-cell voltage-clamp experiments to quantify the maximum conductance of low-threshold potassium channels in octopus cells (Bal and Oertel, 2001, Figure 5]. However, these studies seem likely to have underestimated the dendritic contribution of this conductance due to imperfect space clamp. To investigate this possibility, we replicated the same experimental conditions using our model (Figure 3). The electrode was included in the simulation at the model's soma. The sodium channel conductance and hyperpolarization-activated mixed-cation conductance were set to 0 to mimic blocking of these conductances during the experimental investigation. Since, during the analysis of the published experimental results, the effect of the electrical properties of the electrode series resistance were removed, the model in turn did not include these effects. A voltage step was completed both with and without the presence of low-threshold potassium conductance. By taking the difference in whole-cell current between these two simulations, and dividing by the size of the voltage step, we computed an estimate of the low-threshold potassium conductance as would be obtained experimentally. The experimental results of this procedure from Bal and Oertel (2001) are shown with the results of the modeling in Figures 3A and B, respectively. When the conductance was placed solely in the soma (Figure 3C), it can be seen that the measured conductance was around 80% of the true value. However, it can also be seen that when the conductance was located solely along the dendrites, the measured conductance was around 20% of the true value.

In summary, the total conductance measured using this experimental method underestimated somatic conductance by around 20% but much more dramatically underestimated dendritic conductance. Since potassium channels have been observed to be present in the dendrites (Oertel et al., 2008), the values acquired experimentally seem likely to have greatly underestimated the total conductance in octopus cells. Given this observation, the values acquired experimentally are used as the total somatic conductance only. The magnitude of conductance in the dendrites remains unmeasured. Each of the four dendrites is chosen to be equipped with 10% of the total somatic conductance (the four dendrites together were equipped with 40% of the dendritic conductance). The influence of the choice of value for this parameter was tested under the tolerance regime described in the “Methods” section.

#### Model simulations reveal an optimum sodium channel density in the axon initial segment that replicates experimental data

The sodium channel conductance level was explored in an attempt to re-create the correct experimental electrophysiology. The location of sodium channels was considered to be the axon initial segment, defined in Figure 2.

**Figure 4:**
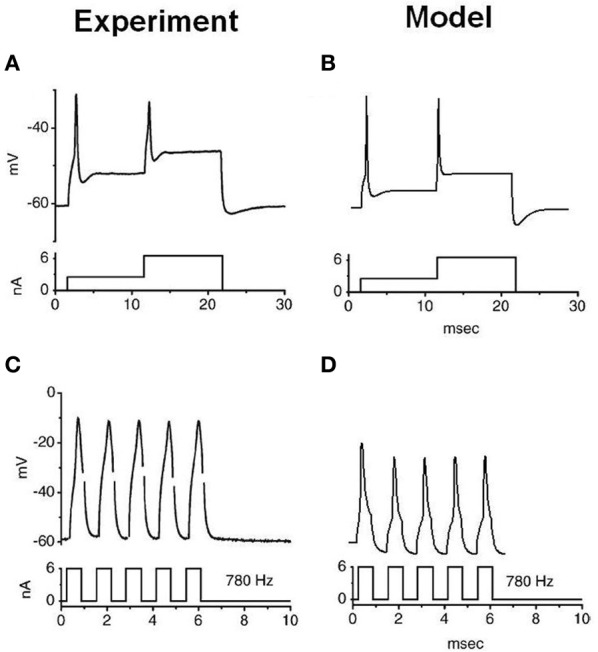
**Experimental and model observations of an octopus cell's membrane voltage when stimulated with step current clamp.** The current stimulus is shown below each plot. **(A,B)** The experimental and model response to stepped current input. **(C,D)** The experimental and model observation of an octopus cell's response to a 6 nA, 780 Hz pulsed current clamp. The results depicted in **(A)** and **(C)** are reproduced, with permission, from Bal and Baydas, (2009, Figure 4), Journal of the Association or Research in Otolaryngology, Am. Physiol. Soc.

**Figure 5:**
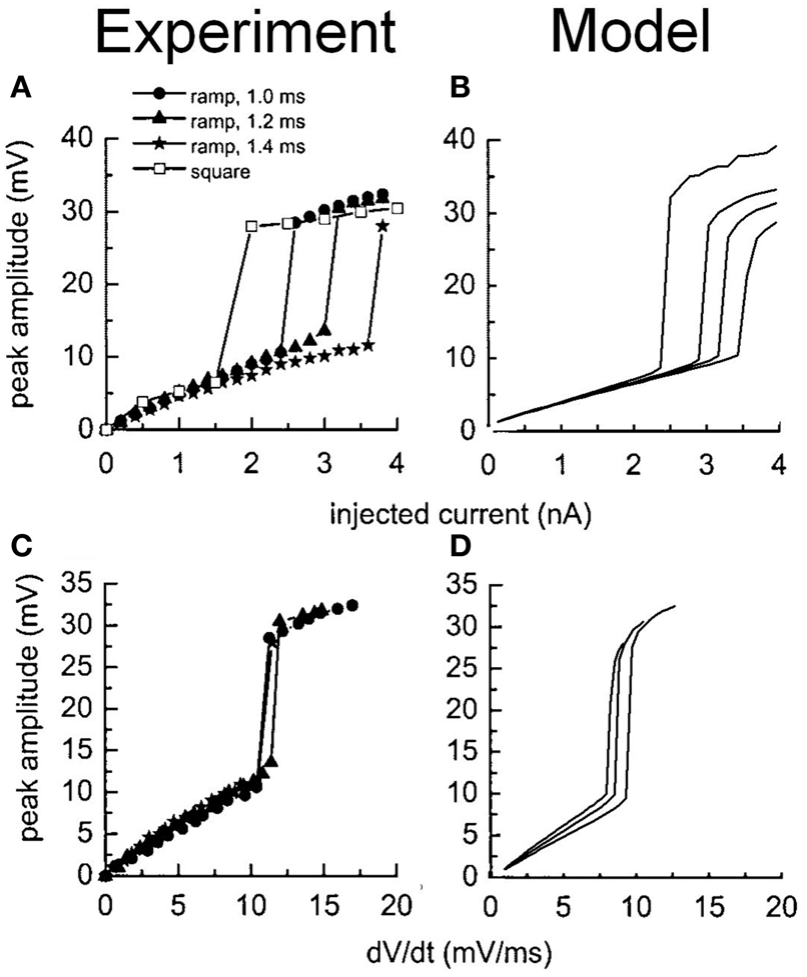
**Experimental and model observations of an octopus cell's membrane voltage when stimulated with ramped current clamp.** The ramp rate and amplitude were varied. **(A)** The peak membrane voltage observed experimentally is plotted as a function of the amplitude of the current clamp. **(B)** The model's response to identical stimuli to **(A)**. **(C)** The peak membrane voltage observed experimentally is plotted as a function of the initial rate of change in the membrane voltage of the cell in response to the current ramp. **(D)** The model's response to identical stimuli to **(C)**. The results depicted in **(A)** and **(C)** are reproduced with permission from Ferragamo and Oertel (2002), Journal of Neurophysiology, Am. Physiol. Soc.

The stimulation protocols were adopted from an experimental investigation into octopus cells (Ferragamo and Oertel, 2002). The total sodium conductance was adjusted to achieve the correct threshold of the rate of change in membrane potential (Figures 5B,D). The same model was then used to re-create the step current clamp experiments originally carried out by Bal and Baydas (2009). The model showed an onset response to simple step currents, and action potentials were produced in response to each new step in a train of current pulses (Figures 4B,D). The experimental results are shown in Figures 4A,C. This model had a rate of change threshold around 9 mV/ms, which fits the experimental results indicating that octopus cells possess a threshold of the rate of change in membrane potential between 5 and 15 mV/ms (Ferragamo and Oertel, 2002).

#### Summary of parameter constraint and verification

Together, Figures 3–5 depict a re-creation of salient experiments performed on octopus cells *in vitro* and completed in previous modeling studies (Cai et al., 2000). These figures demonstrate that the intrinsic electrophysiological properties of the octopus cell model were qualitatively similar to those of real octopus cells.

### Using the model to examine this study's propositions

#### The octopus cell model showed a total dendritic delay of 0.275 ms

Until this point of the investigation the model's temperature was set to 33°C to make comparisons to experimental *in vitro* data. For the purposes of the remainder of the investigation, the temperature was set to 37°C in order to re-create *in vivo* conditions.

**Figure 6:**
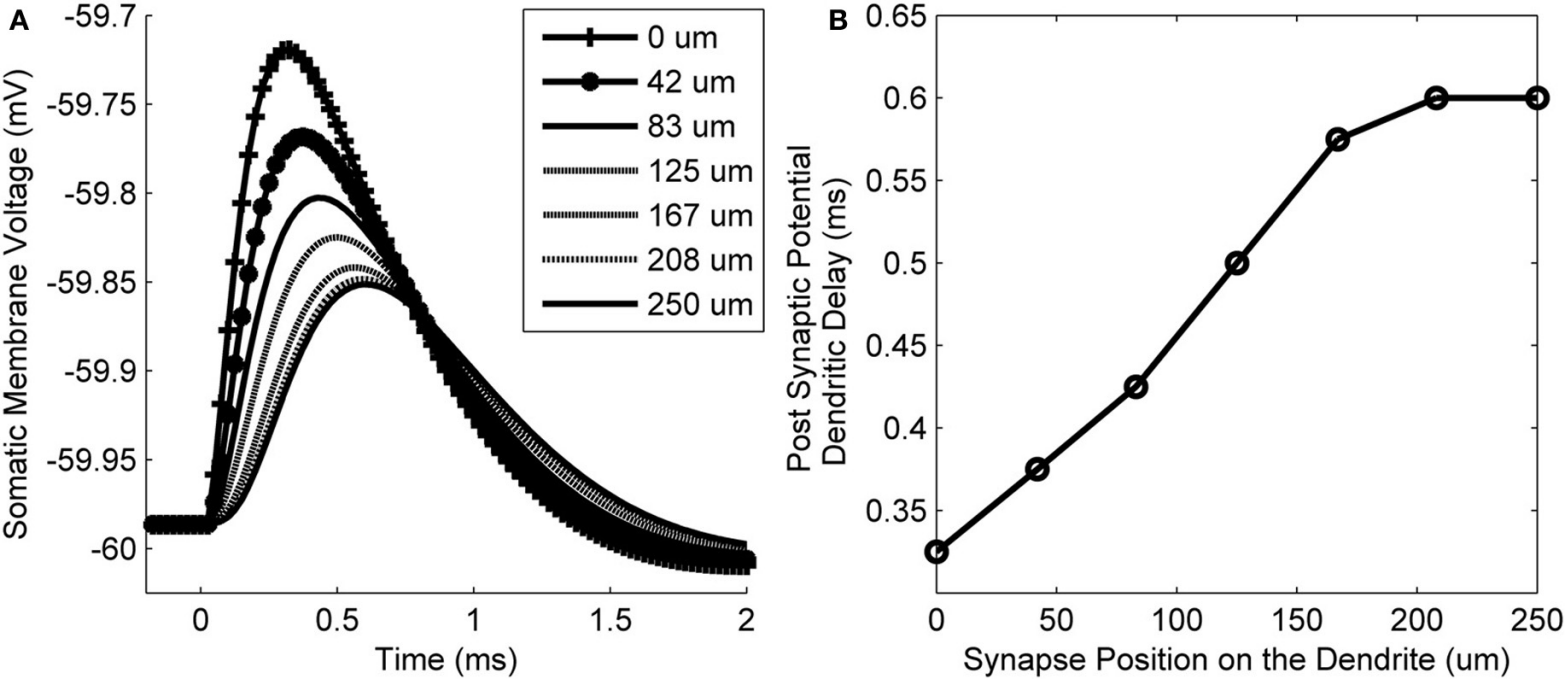
**(A)** The model's membrane voltage at the soma in response to single synaptic inputs. Each curve is associated with input from synapses that were at different locations along the dendrites. The legend shows the longitudinal location of each synapse. **(B)** The delay of the peak membrane voltage vs. the location of the synapse along the length of the dendrite. The propagation time difference between a proximal and distal synapse is approximately 0.275 ms.

The membrane voltage at the soma resulting from synaptic input at different locations along the length of a dendrite is shown in Figure 6A. The delay of the peak voltage at the soma (measured relative to the time of the synaptic event) is plotted as a function of the longitudinal position of the synapse along the dendrite (Figure 6B). The results show that there is a delay of the PSP along the total length of the dendrite of approximately 0.275 ms. This value is referred to as the dendritic delay.

This simulation was repeated with a variety of electrophysiological and morphological conditions (Table 6). The delays represent the capacity of the model's dendrites to compensate for input synapse asynchrony. As expected, a reduction in the dendrite's width or an increase in its length increases the dendritic delay. An increase in the dendritic density of either the low-threshold potassium channels or hyperpolarization-activated mixed-cation channels has little effect on the delay at the nominal densities tested (although see Figure 8).

**Table 6:** **Dendritic delay under various model conditions**.

**Adjusted parameter (original value shown in parentheses)**	**Dendritic delay (ms) (±0.013 ms)**
Nominal parameter values	0.275
Synaptic weights: 4 nS (2 nS)	0.275
Synaptic weights: 1 nS (2 nS)	0.275
Dendrite width: 1.5 μm (3 μm)	0.375
Dendrite width: 6 μm (3 μm)	0.200
Dendrite length: 125 μm (250 μm)	0.100
Dendrite length: 500 μm (250 μm)	0.600
Dendritic *I*_*h*_ conductance: 0 mS/cm^2^ (0.6 mS/cm^2^)	0.275
Dendritic *I*_*h*_ conductance: 1.2 mS/cm^2^ (0.6 mS/cm^2^)	0.275
Dendritic *K*_LT_ conductance: 0 mS/cm^2^ (2.7 mS/cm^2^)	0.300
Dendritic *K*_LT_ conductance: 5.4 mS/cm^2^ (2.7 mS/cm^2^)	0.275
Passive dendrite	0.300

The first row “Nominal parameter values” shows the dendritic delay with parameter values set to those listed in Tables 2, 3. Subsequent entries show the model's dendritic delay with a particular parameter adjusted to a different value (the value in brackets in the first column shows the nominal value of that parameter). The uncertainty in dendritic delay is due to the time-step of the modeling.

**Figure 7:**
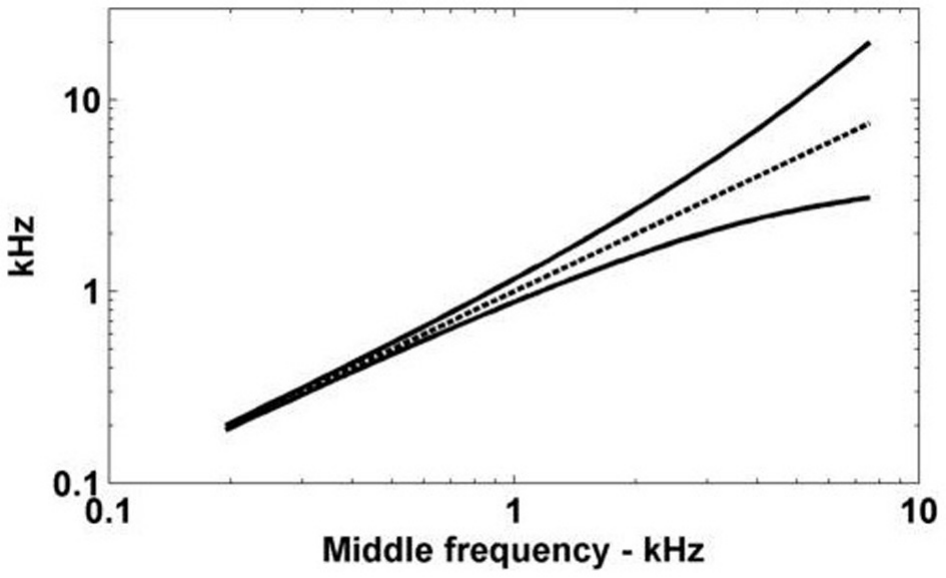
**The spread of ANF CFs with a differential delay of 0.275 ms.** The ANFs with CFs represented by the solid lines have traveling wave delays that are different by exactly 0.275 ms. The horizontal axis gives the log-space center frequency (also shown by the dashed line).

Having quantified the dendritic delay it became possible to determine the theoretical capacity of the dendrites of octopus cells to compensate for the DTWD (Figure 7). This capacity varies depending on the CF of the octopus cell. The cell with a low CF could only compensate for an extremely narrow frequency band of ANF input, however, an octopus cell with a higher CF can receive and compensate for the DTWD associated with a broader frequency band. This observation implies that lower CF octopus cells must either receive a narrower band of ANF input, possess a longer dendritic delay, or do not compensate for input asynchrony. Although Table 5 indicates that this is not surprising, in anatomical studies octopus cells are observed to receive input from lower CF ANFs.

#### Dendritic delay is independent of synaptic strength in the presence of low-threshold potassium channels

**Figure 8:**
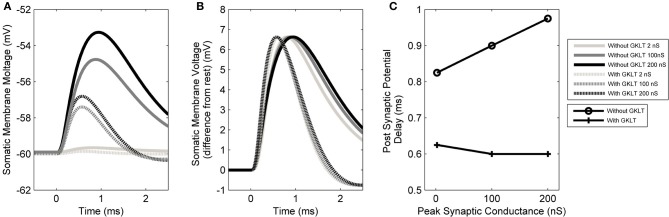
**(A)** The model's membrane voltage at the soma with different synaptic strengths and active conductances. A single synapse at the distal end of the dendrite was activated. The synaptic strength was set to either 2, 100, or 200 nS, representing the strength of 1, 50, or 100 synapses, respectively. The low-threshold potassium conductance was also blocked, then unblocked. In order to maintain the resting voltage of the membrane, the amount of hyperpolarization activated mixed-cation current was adjusted to compensate for the change in low-threshold potassium current (although this was observed to not affect the final outcome). **(B)** The same data normalized so that the PSPs have the same amplitudes as the largest PSP. The vertical axis now shows the membrane voltage deviation from rest so that the change in the form of PSP can be compared directly. This approach is based on the method of Mathews et al. (2010). **(C)** Delay time of the PSP's peak voltage both with and without low-threshold potassium channels.

The form of the PSP in the model was found to be dependent on both the maximum conductance of the synapse as well as the active properties of the soma and dendrite. The PSP at the soma was computed in response to six different parameter configurations: either with or without the low-threshold potassium current, in combination with a synaptic conductance strength of 2, 100, or 200 nS representing the strength of 1, 50, or 100 synapses, respectively (Figures 8A,B). The form of this analysis was inspired by Mathews et al. (2010).

It can be seen that the presence of low-threshold potassium channels has a number of effects. First, there is an increased attenuation of the PSP (Figure 8A). Second, the PSP becomes narrower (see dotted vs. dashed lines Figure 8B). Third, as the amplitude of the synaptic input increases, the PSP's width stays relatively constant (dashed lines Figure 8B). This may enhance the robustness of the model's selectivity for simultaneous synaptic inputs. Finally, and most importantly, it can be observed that the model, without the low-threshold potassium channels, shows an amplitude-dependent dendritic delay (circles in Figure 8C). This delay of around 0.1 ms over the synaptic conductance of 2–200 nS is significant because it is around one third of the total dendritic delay. In the presence of low-threshold potassium channels, the voltage-dependent dendritic delay was absent (crosses in Figure 8C).

#### The octopus cell model responds maximally when the dendritic delay compensates for synaptic asynchrony

In order to investigate the relationship between model cell behavior, dendritic delay and synaptic input asynchrony, it was necessary to compensate for the differential reduction of the PSP peak amplitude due to dendritic filtering. To do this, the most distal synapses were strengthened until the amplitude of their PSP at the soma was the same as that of a proximal synapse. A factor of 4 increase in distal synaptic strength was needed to achieve this. Compensation was applied to all synapses linearly along the length of the dendrite. For example, synapses half way along the dendrite received a factor of 2 increase in their strength. This compensation approach allowed a straightforward investigation of the effects of dendritic delay in isolation. This adjustment was applied to the model for the remainder of the study.

**Figure 9:**
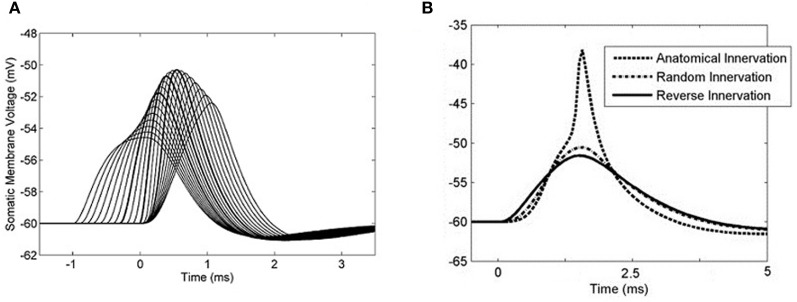
**The somatic membrane voltage responses to different configurations of synaptic input.** Synapses were placed regularly at increasing distances from the soma and were distributed among the four dendrites. A linear delay profile along the dendrite was used as stimulus with a single action potential at each synapse. **(A)** 50 synapses were used and the sodium conductance was set to 0. Input delay profiles were varied between −1 to 1 ms in 0.1 ms increments. A 0.3 ms delay across the inputs produced the largest summed PSP. The PSPs associated with the input −0.3, 0, and 0.3 ms delays are emphasized. **(B)** The sodium channel conductance was re-introduced and in each case 50 synapses were activated, with each synapse activated only once. The synapse activation times were staggered so that the time between the first and last synapse activation was 0.3 ms. The three curves represent three different modes of synaptic innervation: compensated, random, and reversed.

The effects of different artificial synaptic delay profiles were investigated so that an optimum delay profile could be determined. A delay profile was defined as the difference in between spike input time at the most distal synapse and spike input time at the most proximal synapse. With 50 synapses connected along the model cell's 4 dendrites, the sodium channel conductance was set to 0 and the effect of differential input delays could be investigated quantitatively. For each test, each synapse was activated once. Input delay profiles from −1 to 1 ms were used and it was found that the optimum differential delay was 0.3 ms, matching the dendritic delay (Figure 9A). This profile was optimum in the sense that it was this delay profile that produced the highest PSP amplitude. This input profile will be referred to as the optimum input delay profile.

To test the result with action potential production, a simple input event was constructed using 50 synapses distributed in the same way among the four dendrites. Fifty synapses were used because this was the minimum number required to produce an action potential using simultaneous input. These delays were applied with a 0.3 ms input profile in such a way that the dendritic delay compensated for the asynchrony among the synaptic inputs. With this input, the model responded with an action potential (Figure 9B). When synapses with the same range of relative delays (0–0.3 ms) were located randomly along the dendrite, the model did not respond with an action potential. Note that this is not the same as activating the synapses simultaneously. When synapses were located in a reverse anatomical configuration with longer spike latencies associated with synapses located at the distal end of the dendrite, the total PSP became even less depolarized at its maximum point.

Combining these observations, it becomes possible to make a qualitative but strong statement about octopus cells: when the dendritic delay provides compensation for input synapse asynchrony, octopus cells are more sensitive to compensated asynchronous synaptic input than to synchronous synaptic input.

#### Model simulations indicate that, *in vivo*, a non-optimum input delay profile leads to weak response to tones

**Figure 10:**
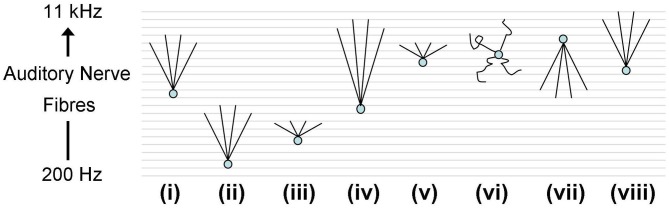
**Octopus cell schematics depicting the different ANF input configurations investigated.** The ANFs are spaced logarithmically in frequency to match the scenario *in vivo*. The changing shapes of the schematics do not represent changes in the dendrite shape or length, rather they represent changes in the ANF innervation profile. (i) The dendritic delay matches and compensated for the DTWD. The span of ANFs received is 2.5–5 kHz. Model “(i)” is the one used in Figures 12A, 14D. (ii) The span is of similar magnitude to that used in (i) but with a lower CF; fibers between 250 Hz and 1 kHz were used. As a consequence the dendritic delay was much shorter than the DTWD. (iii) The span of ANF input is reduced to be 645–800 Hz so that the dendritic delay once more matched the DTWD. This was chosen since this is observed to be the region of greatest spike rate of octopus cells *in vivo*. (iv) The CF is similar to that used in (i) but now 1/3 of the auditory nerve's tonotopicity is used as input (1–10 kHz). In this case the DTWD is longer than the dendritic delay. (v) The CF is similar to that used in (i) but now a much narrower section of the auditory nerve's tonotopicity is used as input (3–3.3 kHz), so that the DTWD is briefer than the dendritic delay. (vi) The octopus cell model receives input randomly along its dendrites although it still receives input from the same range of frequencies as those marked “(i)” and “(vii).” (vii) The bandwidth of innervation is the same as that used in (i) but the innervation profile is reversed so that the dendritic delay adds to (rather than subtracts from) the DTWD. (viii) Very similar to (i), the dendritic delay matches the magnitude of the DTWD and compensates for it. The span of ANFs received is 5.75–11 kHz. This configuration is used in Figure 13 to compare to *in vivo* data.

ANFs from the realistic periphery model (Zilany et al., 2009) were then used as synaptic input. A number of different auditory nerve connectivity configurations were tested to investigate the effect of changes of the octopus cell model CF and the input ANF bandwidth (Figure 10). The aim is not to find the best configuration, but to explore the behavior of a variety of configurations *in vivo*. Although many of these configurations can immediately be excluded from consideration due to their failure to provide an optimum input delay profile, it is interesting to see how a non-optimum input delay profile plays out with realistic input. The ANFs, with logarithmically spread CFs, were spaced linearly along the dendrite.

**Figure 11:**
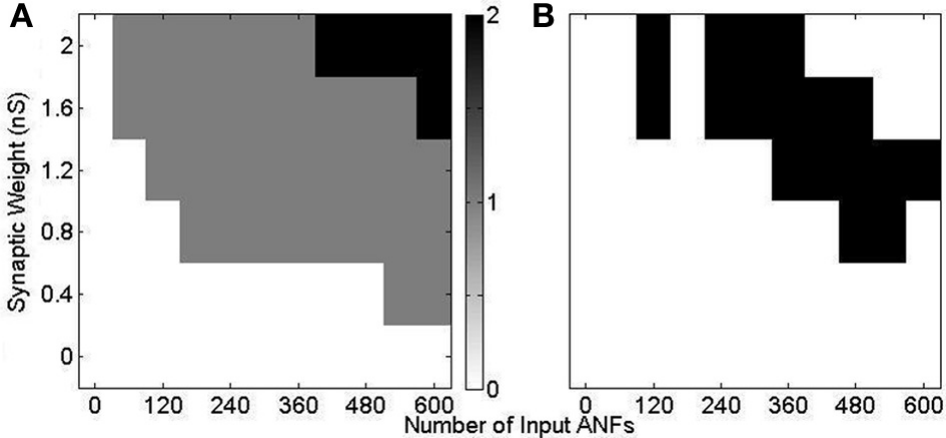
**Response of model configuration (i) in response to a 3 kHz tone of 25 ms duration with 2.5 ms ramp onset and offset.** The synaptic weight and number were varied and are shown on the axes. **(A)** The number of spikes in response to a tone of 70 dB. There is no response with low synaptic weight and number, and 2 action potentials in response to high synaptic weight and number. **(B)** The response to 3 kHz tones of intensity 50, 55, 60, 65, and 70 dB are calculated, as in **(A)**. The black region shows the input drive for which there is a response of one action potential to all 5 tones.

Using model configuration (i) (Figure 10) a range of synaptic weights and numbers were tested (Figure 11). Synaptic weights were varied in increments of 0.4 nS between 0 and 2 nS, corresponding to the experimentally measured synaptic weights. Synaptic number was varied between 60 and 600 (in increments of 60). Tones of 3 kHz and intensity between 50 and 70 dB in 5 dB increments were used as stimulus. The range of observed responses were measured in the number of action potentials. It was found that, with the chosen range of synaptic drive, the 70 dB tone resulted in a response of 0, 1, or 2 action potentials (Figure 11A). This process was repeated with the other tone intensities. Combinations of synaptic weight and number that produced 1 action potential in response to all tone intensities between 50 and 70 dB are marked (Figure 11B). The combination 2 nS/300 ANFs was chosen. Although higher numbers of ANFs may have allowed for smaller synaptic weight, it was desirable to keep the number of ANFs low, partly for computational efficiency.

**Figure 12:**
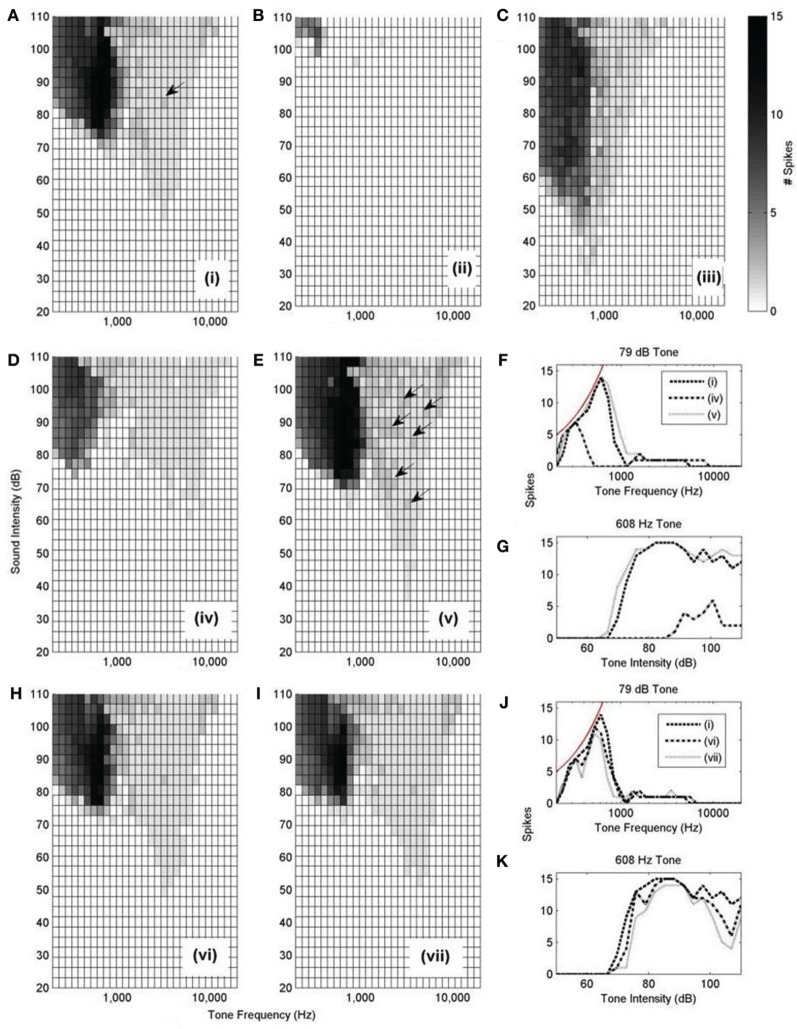
**(A–E,H, and I):** Model responses to tones of different frequencies and intensities. In all cases, 300 ANFs were used as input. The innervation profile is indicated by the roman numeral designation, as depicted in Figure 10. The arrows highlight the presence of double action potentials in relevant sub-figures. The shaded bar shows the number of spikes recorded in response to the 25 ms tone with 2.5 ms ramp onset and offset. A response of 12 spikes corresponds to 480 Hz. However, a response of 1 spike does not necessarily correspond to 40 Hz but rather is observed as an onset response (like that shown in Figure 13B). **(F)** Spike count of each model configuration in response to 79 dB tones of varying frequency. The thin line shows the expected spike rate of a model which responds with perfect entrainment to the presented tone. **(G)** Spike rate of each model in response to tones of 608 Hz of varying intensity. **(J,K)** are similarly constructed to **(F,G)**.

The rate-level response curves of the model under each input configuration (Figure 10 i–vii) were calculated with this synaptic drive (Figure 12). Each of the plots is constructed by varying the frequency and intensity of tones used as auditory stimulus. The number of spikes in response to the 25 ms tone is shown as a different shade of gray.

Model configuration (i) (Figure 12A) showed a bimodal response. This configuration showed a response to tones at a level of approximately 50 dB. Model configuration (ii) (Figure 12B), with a lower CF but the same breadth of frequency input, lacked an optimum input delay profile and showed a significantly weaker response to tones. Figure 12C [configuration (iii)] shows response to low intensity, low frequency tones and may correspond to experimentally observed intensity-frequency response types. However, its input band is extremely narrow, much less that the anatomically observed breadth.

The input bandwidth was extended to cover 1/3 of the tonotopicity of the auditory nerve [configuration (iv)] and also reduced to cover the minimum range that would still contain 300 ANFs [configuration (v)]. It can be seen that configuration (iv) does not entrain to low frequency tones nearly as well as the other investigated configurations (Figures 12F,G). It can also be seen that configuration (v) produces many more double action potentials in response to high frequency tones (Figure 12E). Double action potentials at the onset in the model are undesirable because this behavior is not seen in octopus cells. This makes configuration (v) less likely than configuration (i). However, configuration (v) does entrain with greater reliability to tones than configuration (i) (Figures 12F,G). To test whether the synaptic drive could be reduced to prevent the double action potentials, the synaptic drive was reduced until the double action potentials in configuration (v) were no longer present. However, with reduced synaptic drive, the model's tone intensity thresholds for both configurations (i) and (v) were also found to be increased. In addition to this result, it should also be noted that configuration (v) did not receive optimum input delay profile defined by analysis of Figure 9A. Although this delay is a result of the periphery model, the response to the artificial delay is highly relevant.

Configurations (vi) and (vii) show little difference to configuration (i) in terms of frequency intensity response but are investigated in Figure 14. Note that, given that they do not receive input with the optimum delay profile (as shown in Figure 9), we should not expect them to be acceptable when subjected to finer analysis.

Here, only one of the frequency-intensity response curves, Figure 12A, [configuration (i)], matches the known form of frequency-intensity curves measured from real octopus cells (Godfrey et al., 1975); this was the configuration with a broad and higher frequency ANF innervation configuration [Figure 10 (i)]. However, the broad and high frequency innervation leads to entrainment to low frequency tones and an onset (phasic) response to high frequency tones. From these results, it can be concluded that the most influential input to octopus cells is likely to come from ANFs with CFs above 2 kHz. We do not claim that configuration (i) is “optimum,” rather, of those tested, and without readjusting total synaptic drive, it seems to provide the most realistic response.

The total synaptic drive of 2 nS and 300 ANFs was not readjusted for each new model cell configuration. However, even without this readjustment, Figures 12B,D corresponding to model cell configurations (ii) and (iv) provide extra confirmation of what was already clear from Figure 9; the model cell's sensitivity to input is optimum when dendritic delay matches and compensates for input asynchrony.

**Figure 13:**
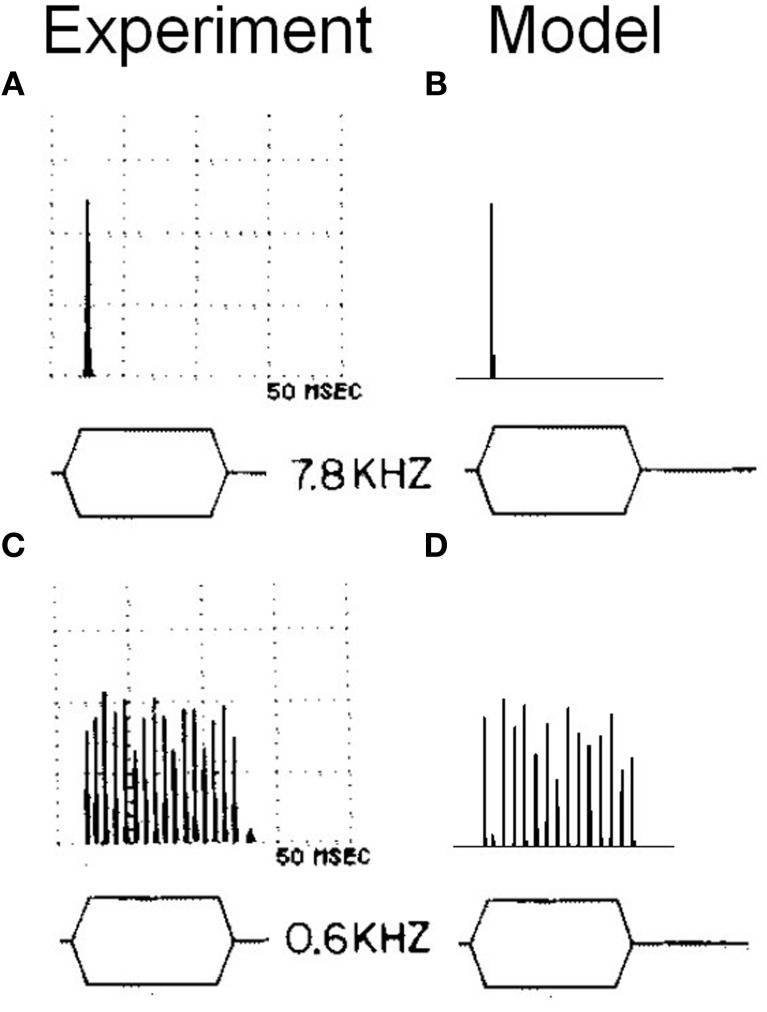
**(A)** Somatic membrane voltage of an octopus cell *in vivo* in response to a 90 dB tone at 7.8 kHz. The results are shown in the form of a post stimulus time histogram in which the stimulus is repeated 50 times and the spiking behavior of the octopus cell model is shown as a histogram. The vertical scales are arbitrary. However, the results depicted are sufficient to demonstrate the bimodal response quality of octopus cells. **(B)** Model (viii) response to an identical stimulus. **(C)** The experimental results with a tone of 0.6 kHz. **(D)** The model's response to the same stimulus as that in **(C)**. The experimental results depicted in **(A)** and **(C)** are reproduced from Godfrey et al. (1975), Journal of Comparative Neurology, John Wiley and Sons, used with permission.

To examine more carefully the *in vivo* behavior of the octopus cell model, the response of a model with input span 5.75–11 kHz was investigated (configuration viii). Configuration (viii) is intended to be similar to configuration (i), but with ANF CF shifted to allow comparison with the chosen experimental data. Tones of 600 Hz and 7.8 kHz were used and the response calculated (Figure 13). The model was found to entrain to the 600 Hz tone and showed an onset response to the 7.8 kHz tone. This bimodal response to sound is the same as that demonstrated in published experimental results (Godfrey et al., 1975).

#### Dendritic delay increases the octopus cell model's sensitivity to sound by compensating for the cochlear traveling wave delay

The effect of dendritic delay was examined more directly by adjusting the pattern of auditory nerve innervation along the dendrite. The rate-level response curves (Figures 12H–K) of the model with random or reversed ANF innervation [configurations (vi) and (vii)] indicate that there is an improvement in the model's sensitivity for configuration (i) over either configuration (vi) or (vii) (Figures 12J,K).

**Figure 14:**
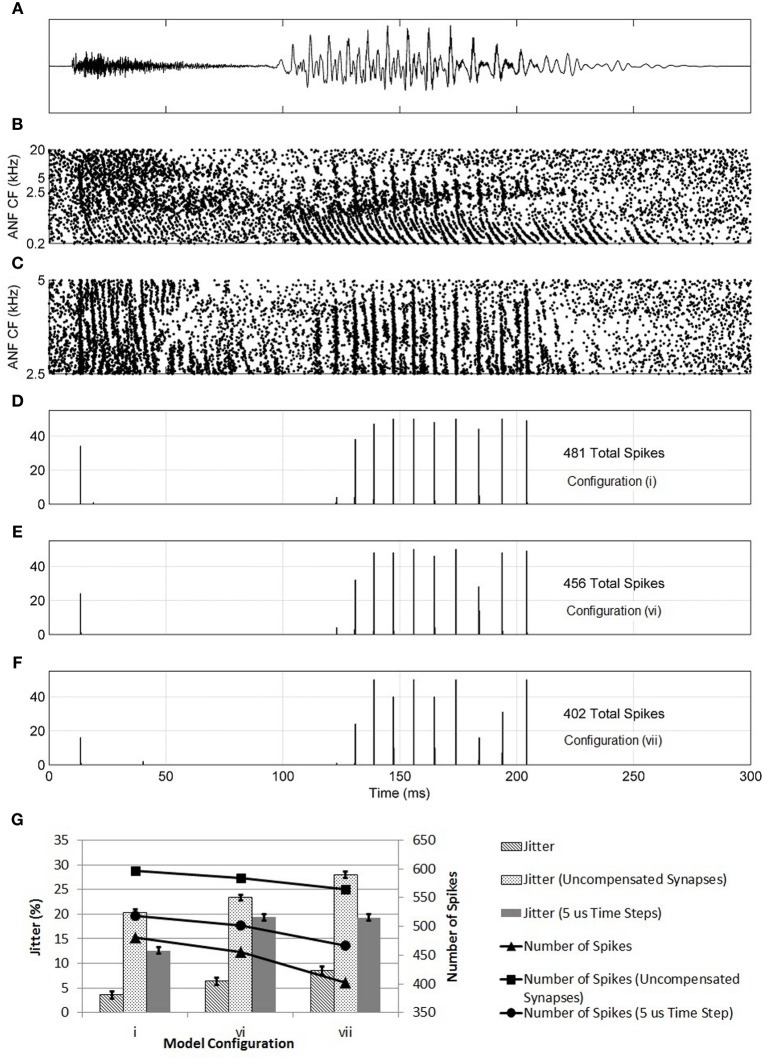
**(A)** The sound intensity of a male vocalization (“twee”) with an amplitude of 67 dB SPL. **(B)** The spiking activity of ANF fibers as calculated by the periphery model across the full tonotopicity. **(C)** The spiking activity of ANF fibers as calculated by the periphery model. Only the fibers that provide synaptic input to the octopus cell model are shown. **(D–F)** The response of the model to the stimulus shown in **(A,B)**. This response is shown in the form of a post stimulus time histogram. The ANF behavior is re-computed using the auditory periphery model 50 times. The resulting spike behavior of the octopus cell model is recorded, and the results shown in the form of a histogram. **(D)** The synapses were placed on the dendrite so that the dendritic delay compensates for the DTWD (model i) **(E)** The synapses were now placed randomly along the dendrites (model vi). **(F)** The synaptic innervation was now reversed from that used in **(C)** so that the dendritic delay adds to the DTWD (model vii). **(G)** The columns show the spike-jitter associated with each model configuration, derived from **(D–F)** corresponding to model configurations (i), (vi), and (vii). The connected marks show the number of spikes. For each configuration the values are shown for constant valued synapses (set at 5 nS) and with compensated synapses but with a model time step of 5 μs. The spike-jitter values are calculated as the percentage of spikes that occur outside the bin with the highest number of spikes for each consonant or glottal pulse. The error bars arise from the assumption that the error in each bar of **(D–F)** is ±1 action potential.

Further evidence of the superiority of configuration (i) was found in the model's response to the speech fragment “twee” (Figure 14). It was found that, with configuration (i), the model responded with an action potential to the onset of the consonant “t” and with further action potentials in response to the glottal pulses of the vowel sound “ee.” With the synaptic innervation randomized or reversed [configurations (vi) and (vii)], the response deteriorated so that fewer action potentials were produced. The reduction in sensitivity, as measured by the number of action potentials (shown in the figure), of a model with a reversed profile of synaptic connectivity was around 25%. It can also be seen in Figure 14 that, as the profile of innervation along the dendrite was changed between configurations (i), (vi), and (vii), the timing jitter of each action potential increased. Specifically, in Figure 14F, many of the peaks in the histogram are spread over two bins to a greater degree than seen in Figure 14D. In addition, many of the spikes are in response to noise rather than the consonant or glottal pulses. Both of these effects are quantified by a measure of spike-jitter. The spike-jitter values are calculated as the percentage of spikes that occur outside the bin with the highest number of spikes for each consonant or glottal pulse. This observation is quantified in Figure 14G. Both the spike-jitter and the total number of pulses showed deterioration when the model configuration was changed to either random synaptic input or reversed synaptic input.

At this stage, it was also possible to test the effects of setting all synapses to the same value rather than assuming that their strength increased along the dendrite in compensation for attenuation due to dendritic filtering. The synapses were set to 5 nS, the same as a compensated synapse half way along the dendrite. The three model configurations investigated in Figure 14 were again tested with the speech fragment “twee.” The number of spikes produced increased because of the increase in strength of the proximal synapses. However, the trend of deterioration from model (i) to (vi) to (vii) was maintained (Figure 14G). It can be concluded that synaptic weight compensation is a factor in reducing spike-jitter. This seems likely to be because, without the contribution of distal synapses, fewer synapses contribute to the production of action potentials. The synapses were also set to the lower value of 1.5 nS, with compensation along the dendrite. The total synaptic drive was maintained by increasing the number of ANFs by 25% to 375. Under this configuration, the same trend of deterioration from model (i) to (vi) to (vii) was observed (results not shown).

The model time step was reduced to 5 μs and the same process repeated (Figure 14G). With a smaller time step, the spike-jitter increased; however, this absolute change is not important for the conclusions of this study. More important is the fact that the same trend is maintained where configuration (i) has a lower spike-jitter than either configuration (vi) or (vii), indicating that the model time step is not a factor in these results.

### Tolerance of the model's behaviour to parameter variation

#### The octopus cell model is most sensitive to changes in temperature and total sodium channel conductance

The sensitivity of the model dynamics to changes in certain parameters was explored. Each parameter listed in Tables 2, 3 was varied and most did not result in significant changes in the model's behavior. However, it was found that the magnitude of high-threshold potassium conductance in the soma could be reduced to zero without any effect on the behavior of the model.

It was also discovered that the model was particularly sensitive to changes in the temperature: an increase of 10% reduced the amplitude of the action potentials and also the likelihood of action potential production in response to stepped current clamp input, while the entrainment to pulsed current train was maintained. A reduction in temperature by 10% increased the size of the action potentials and interfered with the model's response to the pulsed current input, causing a reduction in the number of action potentials. In both cases, the sensitivity to the rate of change in the membrane potential was reduced. A reduction in sodium conductance had a similar effect to an increase in temperature. Neither of these two parameters had a significant effect on the dendritic delay. Other modelers who wish to re-create the behavior of this model should take particular care with these two parameters.

## Discussion

Previous Hodgkin-Huxley computational models (Cai et al., 1997; Kipke and Levy, 1997; Levy and Kipke, 1997, 1998; Cai et al., 2000; Hemmert et al., 2005; McGinley et al., 2005) and two simplified models (Kalluri and Delgutte, 2003a,b) have made a number of contributions to the understanding of the behavior of octopus cells. Briefly, these include a re-creation of the onset response, entrainment to amplitude modulated tones, and a dependence of the spike threshold upon the rate of change in the membrane potential. The role of the kinetics of hyperpolarization-activated mixed-cation current and low-threshold potassium current in the creation of onset responses and current-voltage curves has previously been investigated (Cai et al., 1997). The role of their relative strengths in creating a membrane voltage rate of change spike threshold has also been investigated (Cai et al., 2000). These studies used published data for the hyperpolarization-activated channels (McCormick and Pape, 1990; Banks et al., 1993) and the low-threshold potassium channels (Manis and Marx, 1991). By developing a Hodgkin-Huxley model of a typical octopus cell with active dendrites and input from a realistic model of the auditory periphery, the present investigation has contributed several unique observations. In particular, it was possible to investigate the functional role of synaptic connection location along the dendrites of octopus cells.

It is important to note that although the conductances in this model were set to particular values, a population of real octopus cells will possess a variety of parameter values. It is also likely that mammals with different hearing ranges possess octopus cells with different membrane properties. Neither of these two important factors are investigated in this paper but are good topics for further work. The model showed a high sensitivity to temperature. The size and shape of action potentials were affected by a change in temperature, leading to an altered onset response. This observation is in agreement with experimental evidence that suggests that temperature affects active ion channels to a different degree (Cao and Oertel, 2005). It also motivates further experimental investigations conducted at the true body temperature of the animal, rather than at 33°C where the dynamics were found to be quite different. It was found that the model's behavior, as depicted in the figures of this study, did not depend on the presence of high-threshold potassium channels. This does not mean that high-threshold potassium channels should be omitted from consideration. High-threshold potassium channels have been measured in octopus cells and may contribute to some other mechanism of these cells.

The precise location and magnitude of sodium channels in octopus cells remains unknown. Figures 4, 5 indicate that it is feasible for the sodium channels to be all located at the axon initial segment, given the morphology that we have implemented for the soma and axon and the particular model of sodium channel dynamics utilized. Unlike previous studies, in the present study, the magnitude of the conductance of potassium channels and low-threshold mixed-cation channels was set based directly on experimental results, and the maximum conductance of sodium ion channels was used to tune the model to reproduce realistic *in vitro* responses. The choice of sodium channel location is supported by recent experimental evidence from the avian nucleus magnocellularis (Kuba and Ohmori, 2009), which showed that the location of sodium channels in the axon initial segment is a requirement for precise timing of action potentials in response to synaptic input. Similar configurations are found in mammalian retinal ganglion cells (Fried et al., 2009) and pyramidal neurons in the cortex (Kole et al., 2008) [for a review of this topic, see Clark et al. (2009)]. The location of sodium channels in the axon initial segment may be related to accuracy in the timing of action potentials, as suggested by Kuba and Ohmori (2009), although no evidence on this specific suggestion was gathered in the present study.

Our modeling results indicate that the whole-cell current-clamp method used to measure active ion conductances *in vitro* (Bal and Oertel, 2001) are of limited value in measuring conductances located in dendrites. It was discovered that the total dendritic conductance of low-threshold potassium conductance was most likely underestimated (Figure 3C). In fact, when simulated, the experimental method only reliably quantified the channel conductance that was present in the model's soma. This was due to poor space clamp of the dendrites, indicating octopus cells are not electrotonically compact. The density of active ion channels in membrane areas electrotonically remote from the soma may be relevant to the information processing function of octopus cells, so further experimental work to quantify the magnitude of voltage-dependent conductances across the axon, soma, and dendrites would be very useful.

The observation that the presence of low-threshold potassium channels reduces an amplitude-dependent dendritic delay may have important implications for our understanding of the function of any cell type that is thought to be sensitive to the coincidence of its inputs. The finding has not been fully explored in this study and is a topic for future work. This finding is also relevant to other cells with dendritic low-threshold potassium channels, such as the principal cells of the medial superior olive. The effect of synaptic strength and low-threshold potassium channel conductance on the form of the PSP (Figure 8) was inspired by the study of Mathews et al. (2010). The influence of the channel on PSPs was also investigated by Khurana et al. (2011); however, that study made no note of the influence of the low-threshold potassium conductance on the variation in dendritic delay with PSP amplitude.

When a range of high CF ANFs were connected to the octopus cell model's dendritic tree, with lower CF ANFs at the proximal end of the dendrite and higher CF ANFs at the distal end of the dendrite, the response was more sensitive to tones and speech than with reversed synaptic configuration or with randomly located synapses (Figures 9, 12H–K, and 14). The later configurations showed a deterioration in the model's response. In the context of this result, it is interesting to observe that many experiments have shown that dendrites of octopus cells cross the fibers of the auditory nerve with an orientation appropriate to provide compensation for DTWD (Willott and Bross, 1990; Oertel et al., 2000), although not all experiments show this orientation (Osen, 1969; Webster and Trune, 1982). The current investigation focused on the potential for octopus cells to compensate for an artifact of sound information representation introduced by the cochlea. However, this does not preclude the possibility that, in some cases, the dendritic delays of these cells may compensate for asynchrony due to other causes, such as phase differences in some common kinds of sounds.

The observation that there was an increase in spike-jitter from model (i) to (vi), with random connectivity, and from model (vi) to (vii), with reversed connectivity, is important. Octopus cells are known to produce action potentials with very reliable spike timing (Oertel et al., 2000) and, therefore, the observation provides more evidence that dendritic delay may compensate for DTWD. This increase in spike-jitter was also seen when the synaptic weight compensation for attenuation along the dendrite was removed. This increase in spike-jitter shows that the observed trend does not depend on the assumption that synaptic weights compensate for dendritic filtering. It may be that a concentration of ion channels, such as low-threshold potassium channels, varies along the dendrite in order to create the synaptic weight compensation used in this study. Dendritic density gradients of ion channels have been experimentally observed in other neuron types (Johnston and Narayanan, 2008). Other studies, in particular and most recently Branco et al. (2010), have looked at dendritic delay providing a selectivity for a particular asynchrony across input synapses. That study defined an input “velocity” and dealt with velocities of the order of 1–10 μm/ms, while the present study dealt with velocities of the order of around 1000 μm/ms, 100 times higher. This difference in relevant velocity reflects the two different anatomical locations of the neurons investigated as well as their different functional roles.

Under the dendritic delay proposal, the initial configuration of synaptic connectivity is not critical. This is because the dendritic trees of octopus cells, although oriented in a particular direction, appear to spread in a partly random fashion across the systematically located ANFs. This process and others would initially partially randomize the synaptic location of a particular ANF bouton. Subsequent plasticity could then cull the synapses that do not contribute to the responses of octopus cells, i.e., those that do not compensate for the DTWD. Joelson and Schwartz (1998) and Cao and Oertel (2010) show that there are NMDA receptors in octopus cells that could allow for plasticity, possibly only in very young animals.

Our octopus cell models with lower CFs (on the order of hundreds of Hertz) were found to have trouble detecting coincidence across fibers from a broad range of CFs with DTWD amounting to many milliseconds. Octopus cells that receive input from lower CF ANFs may possess a narrower frequency band of ANF input or, if the magnitude of the frequency bandwidth remains fixed, may possess a longer, narrower, or less active dendrite. It should also be noted that octopus cells with this low frequency innervation would also be seen to have a longer first spike latency than previously observed (Golding et al., 1995). Importantly, if the input to octopus cells is restricted to high CF ANFs, this does not prevent the cells from responding to low frequencies. Our model showed that even without input from low frequency fibers, the model responded to high intensity, low frequency tones (Figures 12A,H–K) as observed experimentally (Godfrey et al., 1975; Rhode and Smith, 1986; Rhode et al., 2010). The form and features of the bimodal response curves (Figures 12H–K) match very closely with experimentally observed responses (Godfrey et al., 1975). The proposition that octopus cells receive input mainly from high frequency ANFs is also supported by two experimental findings. First, octopus cells possess a very short first spike latency (Rouiller and Ryugo, 1984) that could not be created by synapses from low CF ANFs that possess a long first spike latency due to the traveling wave delay. Second, octopus cells are generally observed to possess a high CF (see Table 5). When discussing the dominant input frequency band to octopus cells, it is also important to note that high intensity tones are not the only low frequency stimulus that might induce a response in octopus cells. Clicks produce energy across a broad range of frequencies, as do the glottal pulses of speech. The latter can be seen to stimulate the model even with relatively low stimulus intensities (Figure 14).

The afferents from the octopus cell area of the cochlear nucleus are known to project exclusively to the monaural areas of the auditory brainstem [the ventral region of the ventral nucleus of the lateral lemniscus and the superior paraolivary nucleus (Schofield, 1995; Schofield and Cant, 1997; Cant and Benson, 2003)]. This anatomical observation, along with the results of Figure 14, strongly suggest that octopus cells are well suited to providing a finely timed representation of the temporal envelope of natural sounds, including the glottal pulses associated with vocalizations. In humans, this information may be used at the level of the inferior colliculus, thalamus, and cortex to increase the reliability of the classification of different speech sounds. The 25% reduction in sensitivity that occurs without dendritic delay compensation means that octopus cells would be less useful to the higher areas of the brainstem, although the precise detrimental effect depends on the overall role of octopus cells in the auditory brainstem, something that remains an open question.

### Conflict of interest statement

The authors declare that the research was conducted in the absence of any commercial or financial relationships that could be construed as a potential conflict of interest.
